# A nationwide trend analysis in the incidence and mortality of Creutzfeldt–Jakob disease in Japan between 2005 and 2014

**DOI:** 10.1038/s41598-020-72519-0

**Published:** 2020-09-23

**Authors:** Yoshito Nishimura, Ko Harada, Toshihiro Koyama, Hideharu Hagiya, Fumio Otsuka

**Affiliations:** 1 Department of General Medicine, Okayama University Graduate School of Medicine, Dentistry and Pharmaceutical Sciences, 2-5-1 Shikata-cho, Kita-ku, Okayama, 700-8558 Japan; 2 Department of Pharmaceutical Biomedicine, Okayama University Graduate School of Medicine, Dentistry, and Pharmaceutical Sciences, Okayama, 7008558 Japan

**Keywords:** Epidemiology, Health policy, Neurological disorders

## Abstract

In the era of hyper-ageing, Creutzfeldt–Jakob disease (CJD) can become more prevalent as an important cause of dementia. This study aimed to evaluate the trends in crude and age-adjusted CJD-associated mortality and incidence rates in Japan using national vital statistics data on CJD-associated deaths among individuals aged over 50 years, as well as the government-funded nationwide CJD surveillance data (pertaining to the years 2005–2014) in Japan. The data were analysed using the Joinpoint Regression Program to estimate the long-term trends and average annual percentage changes (AAPCs). Overall, the AAPCs of age-adjusted CJD-associated mortality rates rose significantly over the study period (3.2%; 95% confidence interval [CI] 1.4–5.1%). The AAPC of the age-adjusted incidence rates also increased (overall 6.4%; 95% CI 4.7–8.1%). The CJD-associated increases in the mortality and incidence rates were especially prominent among adults over the age of 70 years. Given this trend in aging of population, the disease burden of CJD will continue to increase in severity. Our findings thus recommend that policymakers be aware of the importance of CJD and focus on preparing to address the increasing prevalence of dementia.

## Introduction

Characterized by spongiform encephalopathy that causes rapidly-progressing dementia and a rate of high mortality within a year of the onset of clinical symptoms, Creutzfeldt–Jakob disease (CJD) is the most common human prion disease^1^. CJD is categorized into four different types: variant (vCJD), sporadic (sCJD), familial, and iatrogenic^2^. While the disease first received the public recognition after the emergence of bovine spongiform encephalopathy and the subsequent surge in the global incidence of human vCJD cases between the late 1990s to early 2000s^3,4^, sCJD accounts for approximately 85% of CJD cases^5^. Since prion proteins reportedly accumulate in brain cells as they age^6,7^, sCJD most frequently occurs in late-middle old age^8^. Hence, considering the ageing of the worldwide population, the potential increase in sCJD cases is a pressing global concern. Indeed, by 2030, the global population of individuals aged 60 years or older will increase by 34%^9^. This rise will shift the demographic and epidemiological landscapes of disease.

The global trends of mortality and incidence of CJD are currently trending upwards. For example, the number of sCJD cases in Australia rose from 1970 to 2018^10^. In the United States, the Centers for Disease Control and Prevention reported an increasing trend in CJD-associated deaths, with an average annual rate of 3.5 per million for those over 50 years of age^11,12^. Likewise, the age-standardised mortality rate from sCJD in Canada increased from 1998 (0.84 per million) to 2013 (1.30 per million)^13^.

The World Health Organization (WHO) announced the “Decade of Healthy Aging 2020–2030” as a call for an increased focus of worldwide attention on population ageing^14^. According to the most recent United Nation’s World Population Prospects, Japan has the highest proportion of people aged 65 years or older (28% of the population)^15^. Hosted by Japan in 2019, the Group of 20 (G20) also adopted the issue of population ageing as one of the main themes of the G20 Health Ministers’ Meeting for the first time^16^. In particular, the G20 Health Ministers’ called upon the importance of committing to the development and implementation of a national action plan to address dementia^17^. Because CJD is more prevalent among older individuals who feature an increased vulnerability to progressive dementia, it is imperative to evaluate longitudinal national trends of CJD-associated mortality rates and thereby help to inform robust dementia response action and future strategic policy planning.

While previous studies have reported on the epidemiology of regional variations in CJD and its incidence and mortality in Japan^18–20^, no previous study has investigated the long-term trends in CJD-associated mortality and incidence rates in Japan since Doi et al. in 2007^20^. Our study, thus, provides an updated report on the recent national trends in CJD-associated mortality and incidence.

## Results

### The accumulated number of CJD-associated deaths

**Figure 1:**
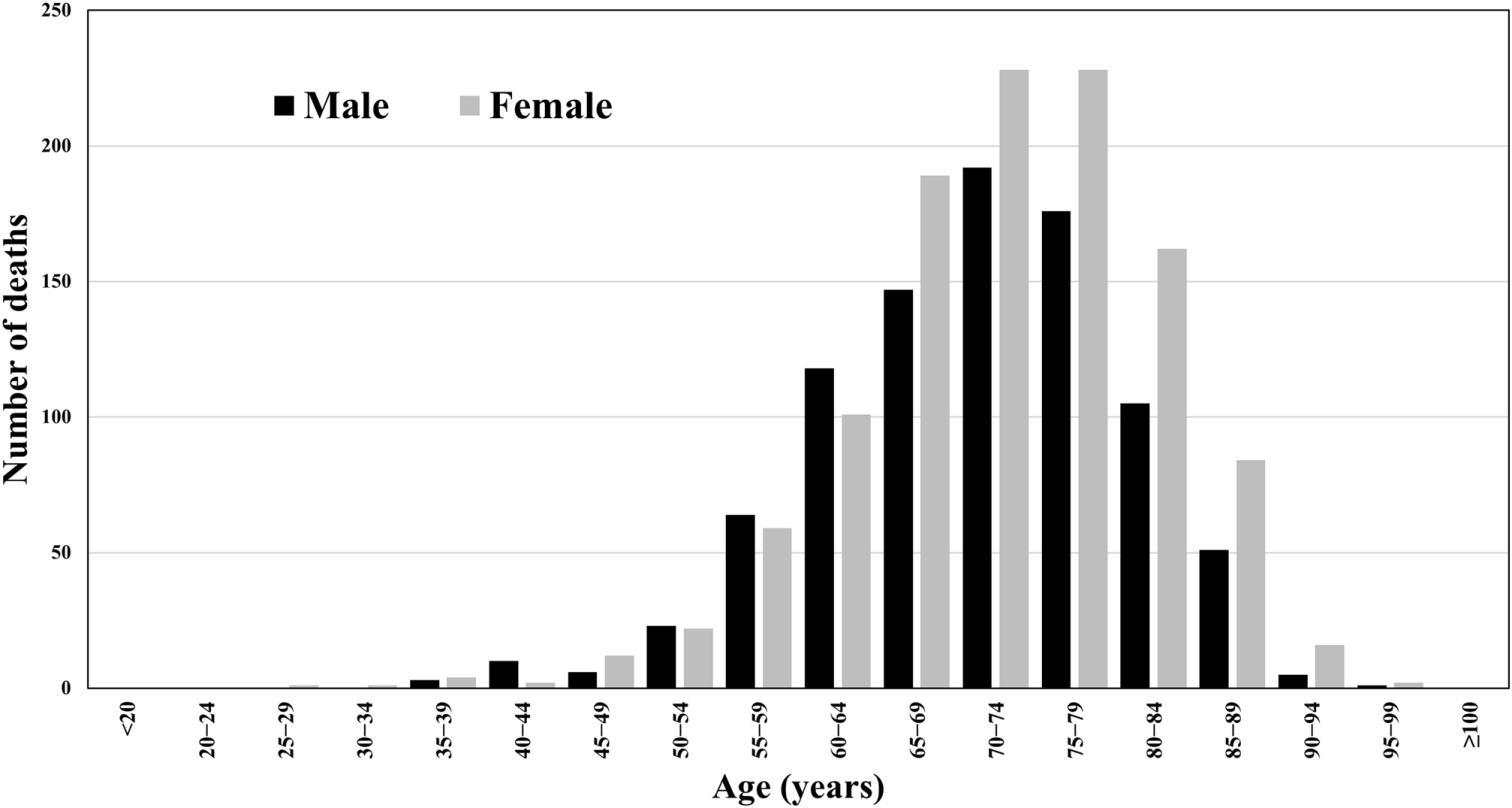
Total number of Creutzfeldt–Jakob Disease-associated death by sex, 2005–2014. The numbers of CJD-associated deaths by five-year age groups stratified by sex are shown. The number of CJD-associated deaths reached its peak in the 70–74 years age group in male (192 deaths over the 10-year period), and in the 70–74 and 75–79 years age groups in female (228 deaths over the 10-year period in both age groups).

A total of 2012 CJD-associated deaths were recorded between 2005 and 2014. Figure 1 shows the number of CJD-associated deaths stratified into 5-year age groups according to sex. The number of CJD-associated deaths rose progressively with age from the age group of 50–54 years; the number peaked in the age group of 70–74 years among men (192 deaths over the 10-year period) and in the age groups of 70–74 and 75–79 years among women (228 deaths over the 10-year period in both age groups).

### Trends in the crude CJD mortality rates according to sex and age

**Figure 2:**
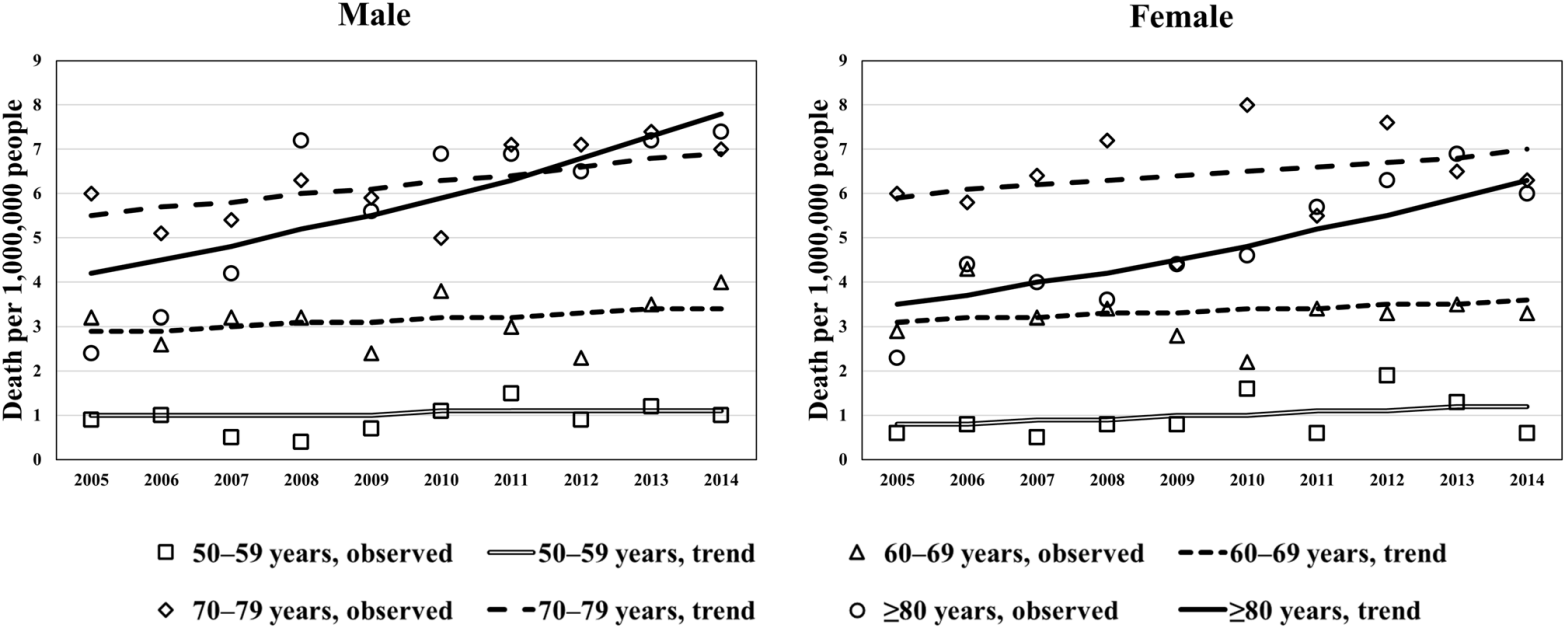
Trends in crude mortality rates (per 1,000,000 population) of Creutzfeldt–Jakob Disease-associated death by age and sex, 2005–2014. The trends in crude mortality rates of CJD-associated deaths in patients over the age of 50 years by 10-year age groups and sex are described. The detailed numerical values for the age groups are available in Supplementary Table S1.

Figure 2 describes the trends in crude mortality rates of CJD-associated deaths in patients over the age of 50 years according to 10-year age groups and sex. The detailed numerical values for the age groups are provided in Supplementary Table S1. The crude mortality rate per 1,000,000 individuals in the age group of ≥ 80 years increased from 2.4 in 2005 to 7.4 in 2014 among men and from 2.3 in 2005 to 6.0 in 2014 among women. In the other age groups, the extents of an increase in the crude mortality rates were small. In the last year of the study, the crude mortality rates reached their highest point in the 10-year study period in the age groups of ≥ 80 years among men and of 70–79 years age among women. The results of the joinpoint regression analysis of the crude mortality rates according to the 10-year age groups are shown in Table 1. Overall and among women, the average annual percentage changes (AAPCs) were stable in all the age groups, except for the age group of ≥ 80 years, with a statistically significant continuous increase across the entire period. Among men, the AAPCs of the crude mortality rates period remained stable in the age groups of 50–59 and 60–69 years across the entire study period, while significant increases were observed in the age groups of 70–79 and 80–89 years.

**Table 1:** Trend analysis in crude and age-adjusted mortality rate of Creutzfeldt–Jakob disease per 100,000 by sex and age, 2005–2014.

Age group (years)	Average APC (%) [95% CI]
**Overall**	
50–59	4.9 [− 1.8, 12.0]
60–69	0.8 [− 1.8, 3.4]
70–79	2.2 [− 0.2, 4.6]
**≥ **80	8.8* [5.7, 11.9]
**Male**	
50–59	2.3 [− 4.8, 9.9]
60–69	2.0 [− 2.4, 6.6]
70–79	3.5* [0.7, 6.3]
**≥ **80	13.6* [8.4, 19.0]
**Female**	
50–59	9.0 [− 2.7, 22.1]
60–69	− 0.5 [− 4.6, 3.9]
70–79	1.1 [− 3.1, 5.6]
**≥ **80	8.8* [4.9, 12.9]

*AAR* age-adjusted rate, *APC* annual percentage change, *CI* confidence interval.

*Significantly different from zero (*p* < 0.05).

### Trends in the age-adjusted mortality rates of CJD by sex

**Figure 3:**
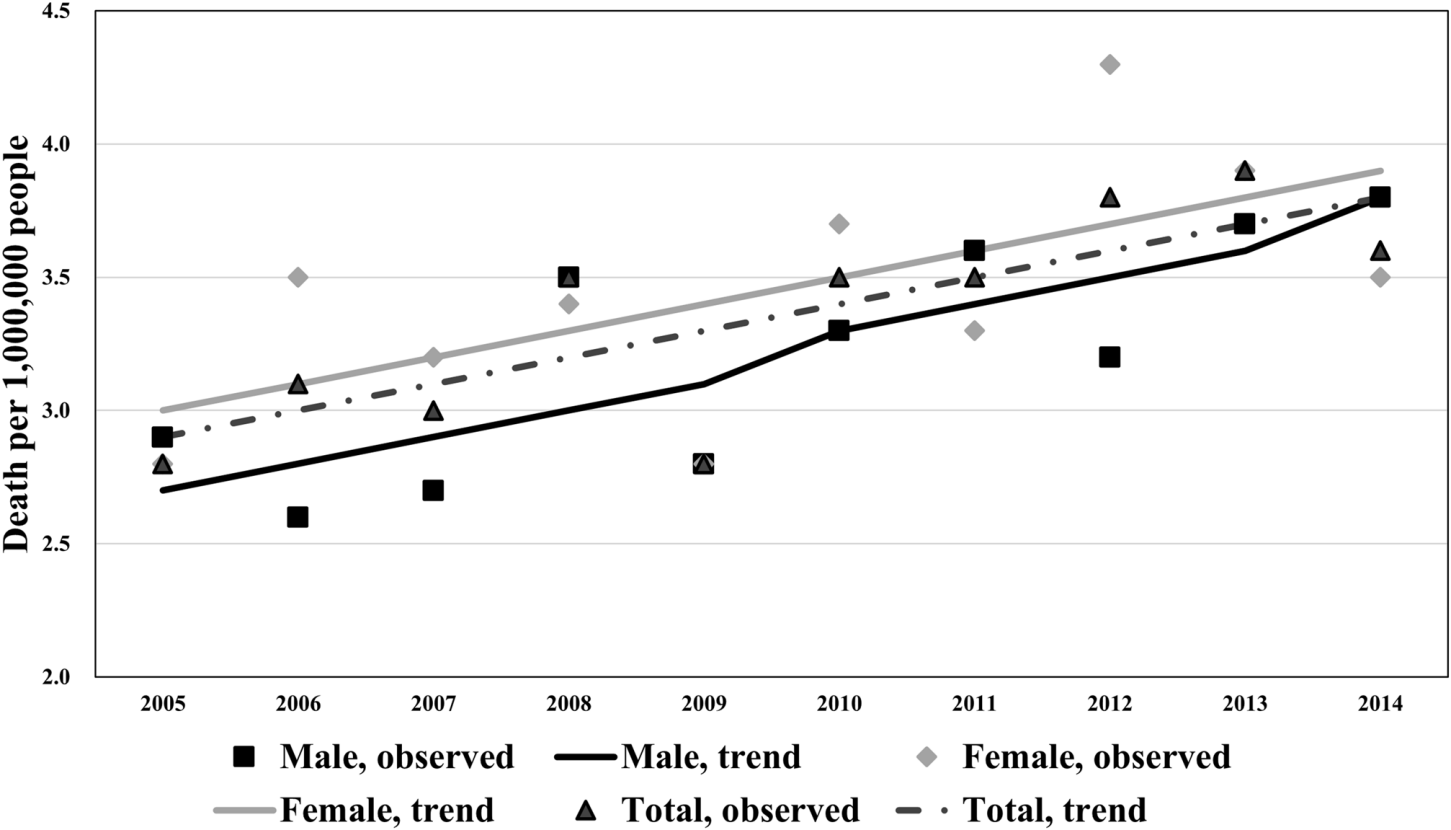
Trends in age-adjusted rates of Creutzfeldt–Jakob Disease-associated death by sex, 2005–2014. The trends of age-adjusted CJD mortality rates in the entire period by sex are shown. Average annual percentage change (%) [95% confidence interval] in total, male, and female were 3.2* [1.4, 5.1], 3.7* [1.3, 6.0], and 2.9 [− 0.1, 5.9], respectively. *Significantly different from zero (*p* < 0.05).

The trends in the age-adjusted CJD mortality rates across the entire period are shown in Fig. 3 according to sex along with the results of the joinpoint regression analysis. The age-adjusted CJD-associated mortality rates rose significantly over the study period among men; the increase was continuous but statistically nonsignificant among women.

### CJD incidence by sex

**Figure 4:**
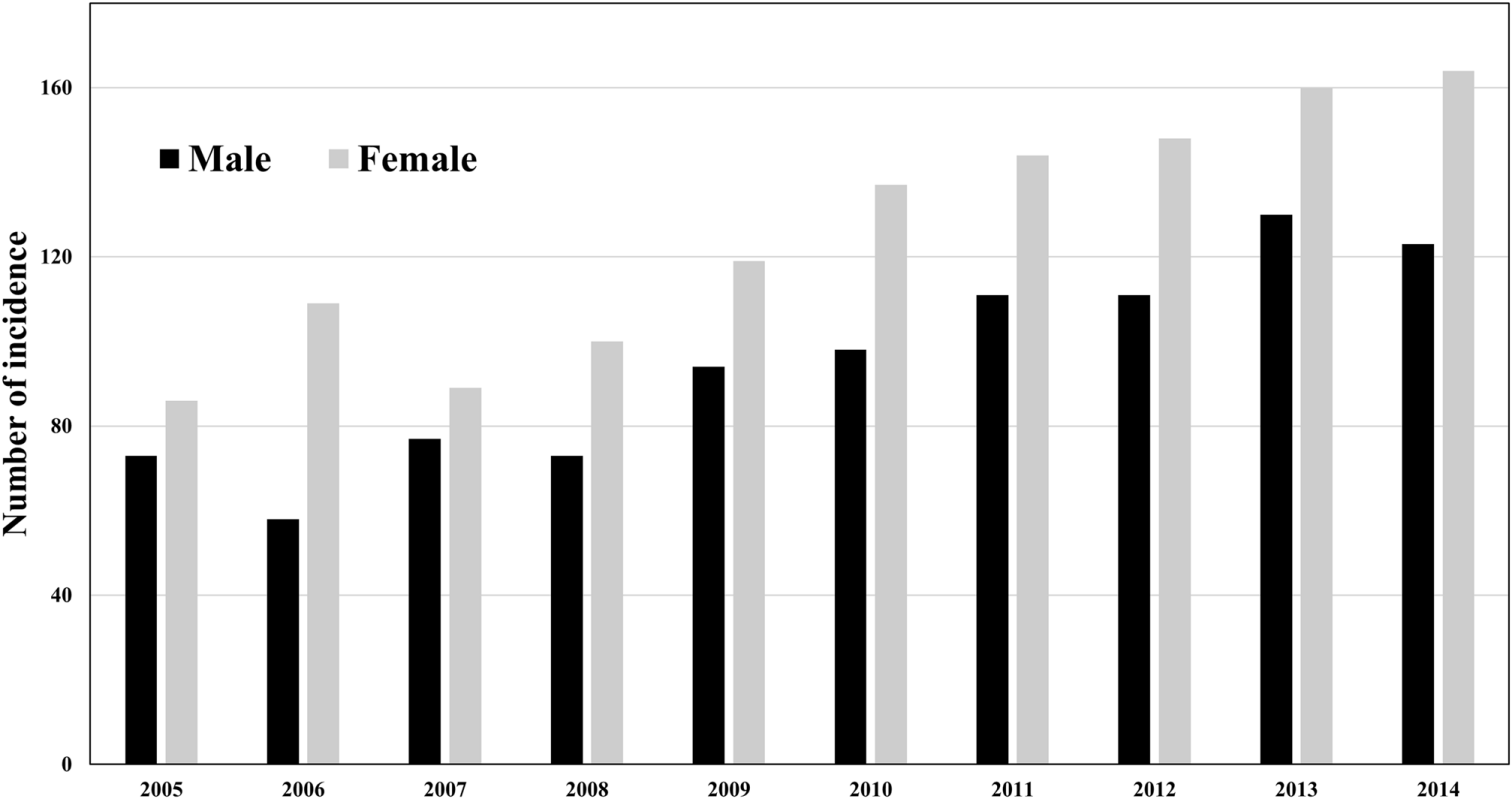
Numbers of Creutzfeldt–Jakob Disease incidence by sex, 2005–2014. The numbers of CJD incidence in each year stratified by sex are shown. The number of CJD incidence continuously increased in females over the entire study period, while it reached a peak in 2013 in males.

Figure 4 shows the CJD incidence rates for each year stratified by sex. The rates of CJD incidence increased continuously among women across the entire study period but reached a peak among men in 2013.

### Trends in the crude CJD incidence rate by age

**Figure 5:**
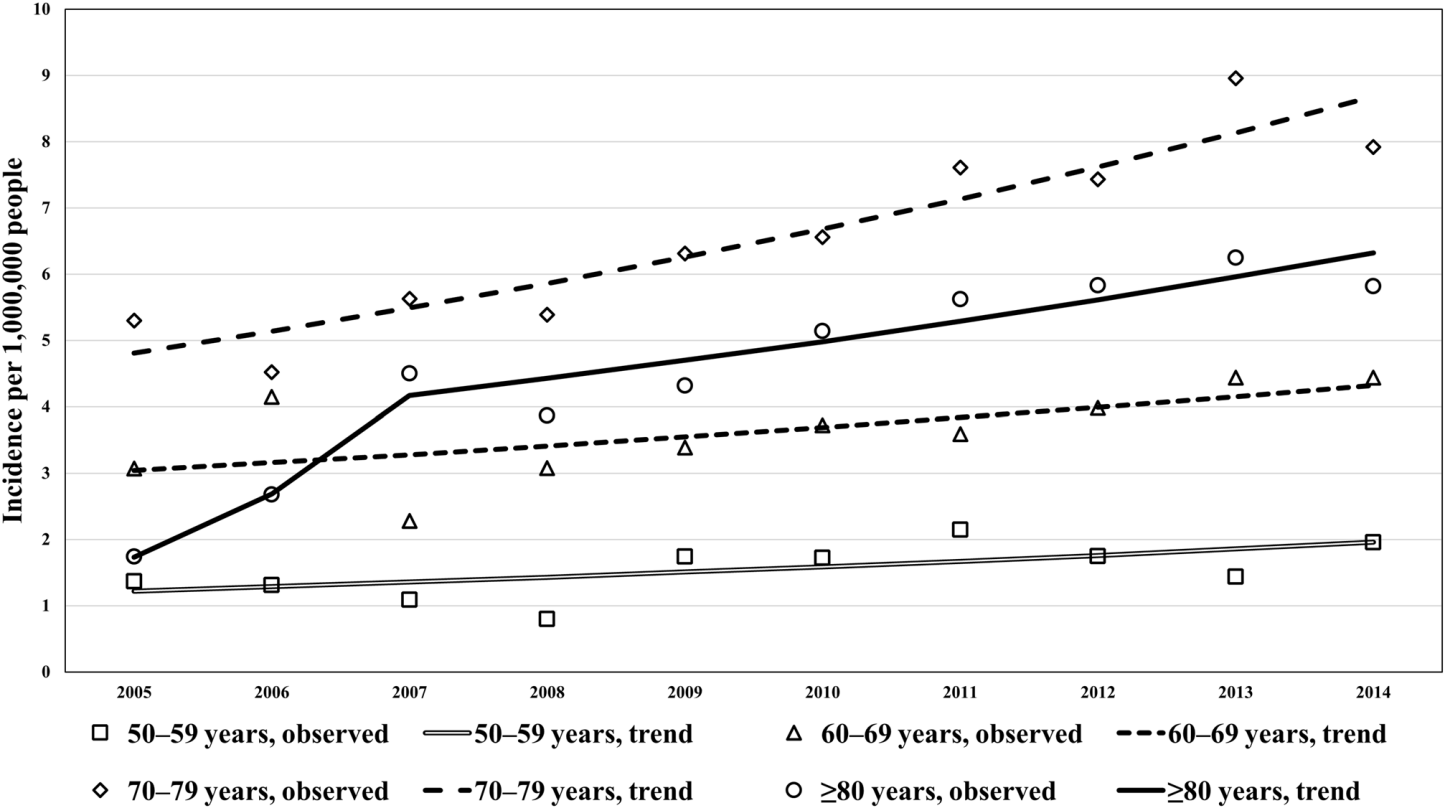
Trends in crude incidence rates (per 1,000,000 population) of Creutzfeldt–Jakob Disease, 2005–2014. The trends in crude incidence rates of CJD over the age of 50 years by 10-year age groups and sex are shown. The crude incidence rates per 1,000,000 population had continuous increases in the 60–69 years age groups or older.

Figure 5 shows the trends in the crude incidence rate of CJD among individuals older than 50 years of age stratified by 10-year age groups and sex. The crude incidence rates per 1,000,000 individuals increased continuously in the age groups of 60–69 years or older. The results of the joinpoint regression analysis of the crude CJD incidence rates are presented in Table 2 according to the 10-year age groups. In the age groups of 60–69 and 70–79 years, AAPCs significantly increased across the entire study period. In the age group of ≥ 80 years, the rise in the APC between 2005 and 2007 (55.2% increase per year) exceeds that between 2007 and 2014 (6.1% increase per year), with a statistically significant, continuous increase across the entire period.

**Table 2:** Trends in the annual crude and age-adjusted incidence rate of Creutzfeldt–Jakob disease per 1,000,000 population aged ≥ 50 years by age group, 2005–2014.

Age group (years)	Period 1		Period 2		Average APC (%), [95% CI]
	Years	APC (%)	Years	APC (%)	
50–59	2005–2014	5.4			5.4 [− 0.5, 11.6]
60–69	2005–2014	4.0			4.0* [0.2, 8.0]
70–79	2005–2014	6.8			6.8* [4.5, 9.1]
≥ 80	2005–2007	55.2	2007–2014	6.1	15.5* [5.3, 26.7]

Within each age group, periods were separated as Period 1 and Period 2 when the trend changes were statistically detected in the joinpoint regression analysis during the study period.

*AAR* age-adjusted rate, *APC* annual percentage change, *CI* confidence interval.

*Significantly different from zero (*p* < 0.05).

### Trends in the age-adjusted CJD incidence rates

**Figure 6:**
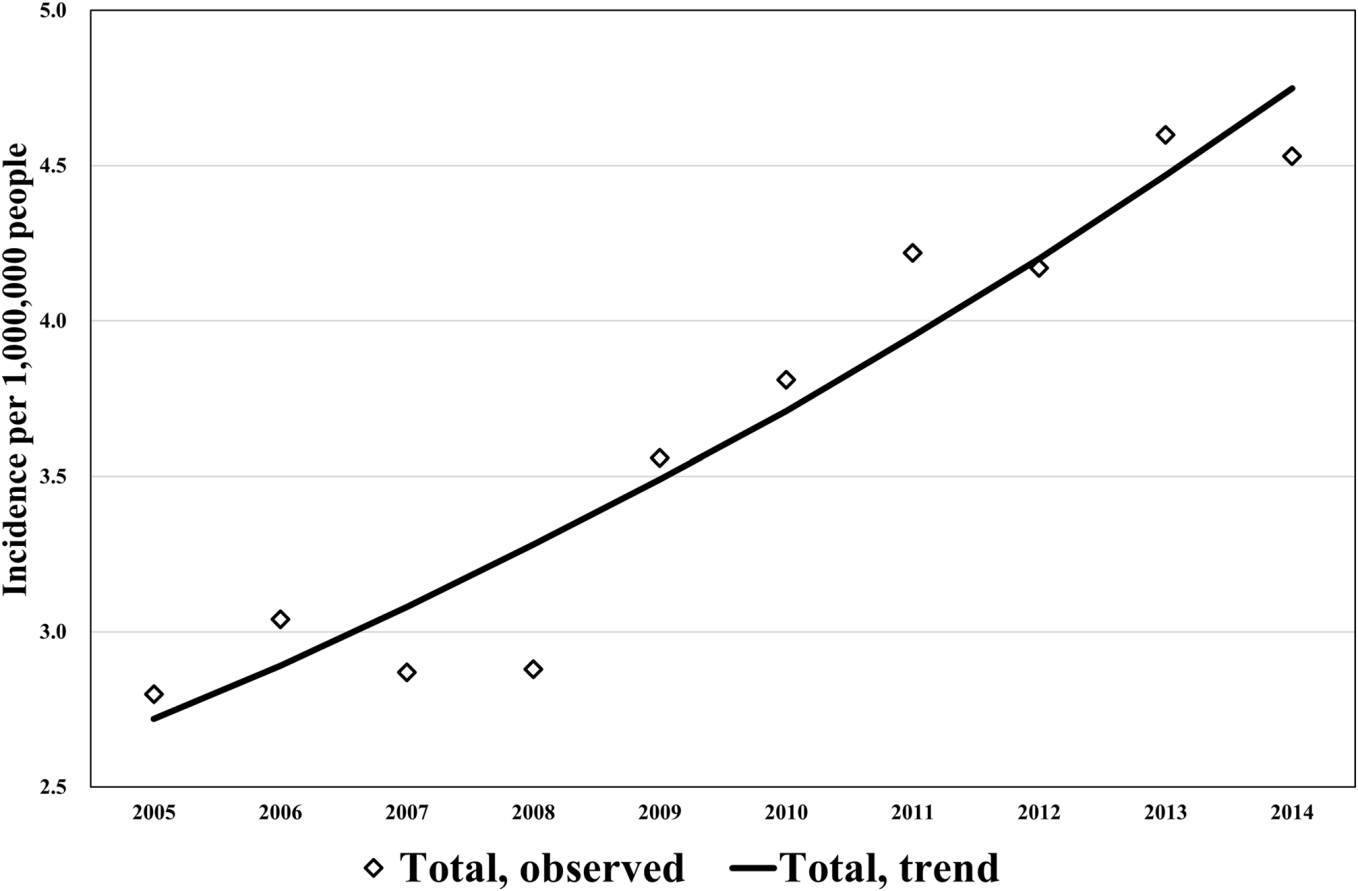
Trends in age-adjusted rates (per 1,000,000) of Creutzfeldt–Jakob Disease incidence, 2005–2014. The trends of the overall age-adjusted CJD incidence rates in the entire period are shown. The overall age-adjusted CJD-associated incidence rates showed a significant increase over the period; average annual percentage change (%) [95% confidence interval]; 6.4* [4.7, 8.1]. *Significantly different from zero (*p* < 0.05).

The trends in the overall age-adjusted CJD incidence rates across the entire period are shown in Fig. 6 along with the results of the joinpoint regression analysis. The overall age-adjusted CJD-associated incidence rates rose significantly over the study period, with an AAPC of 6.4% (95% CI 4.7–8.1).

## Discussion

Our study revealed an increase in the absolute number of deaths, mortality rates, and incidence rates associated with CJD—even after age-adjustment—in Japan between 2005 and 2014. The significantly increasing trends in the CJD-associated mortality and incidence rates were especially prominent in the older age groups. Previous surveillance data and reviews have described that comparable increasing trends in both mortality and incidence of CJD have been observed globally. According to the latest report from the Creutzfeldt–Jakob Disease International Surveillance Network, annual mortality rates of sCJD had risen in the most participating countries from 1993 to 2018^21^. In European countries including the Czech Republic, Slovakia, and the United Kingdom, increases in the sCJD incidence have been reported^22–24^. According to these studies and the current data, it should be noted that Japan might have had higher CJD incidence (more than 4.5 per million in 2014) than other countries. While CJD has been considered a rare disease, the phenomenon of population ageing may trigger a rise in the incidence of CJD and the attendant socioeconomic and healthcare burdens.

According to the previous CJD-associated mortality analysis conducted from 1979 to 2004 in Japan^20^, the crude mortality rate reached a peak of 1.3 per million in 2004. Our analysis indicates that the steep trend has continued, with 4.1 cases of CJD per million accounted for in 2014 (Supplementary Table S1). While the number of CJD-associated deaths was highest in the 70–79 years age group from 1979 to 2004 in Japan, our crude mortality analysis showed a predominant upward trend among individuals of over 80 years of age, indicating a shift in the CJD disease burden to the more aged population. Concerning the age-adjusted mortality, our results show a continuously increasing trend across the study period, with an APC of 3.7% among men and of 2.9% among females. These results agree with those found by Doi et al., with an age-adjusted mortality of 3.1% among men and 3.9% among women. This increasing trend suggests that CJD will soon aggravate the disease burden in the hyper-ageing Japanese society.

The increased CJD-associated mortality rates could be explained by the rise in the rate of CJD incidence. The prognosis of CJD is considered poor, with uniform fatality and a median life expectancy of about 6 months after diagnosis worldwide^25,26^. Hence, as was observed in the present study, the mortality and incidence rates are almost identical. Various factors could account for the increase in the rates of CJD incidence: e.g., increased awareness of the disease^1^, improvement in the accuracy of diagnostic tests^2^, and demographic changes characteristic of a hyper-ageing society. While the upward trends in the rates of mortality and incidence may be partly attributable to improvements in diagnosis, we propose the ageing of the population as an important contributor to the CJD trends in Japan.

According to the Japanese national statistics, the population of individuals of over 65 years of age was 35.58 million in 2018, accounting for 28.1% of the whole population^27^. The Japanese surveillance data indicates that approximately 80% of the CJD patients in Japan were sCJD, which is more common among older individuals^28^. Hence, if the increased CJD-related incidence and mortality are primarily attributable to sCJD, it follows that both rates would rise as the population ages. These observations are compatible with our study results, which suggest that the number of CJD-associated deaths was highest in the age groups of 70–74 and 75–79 years. From a pathophysiological perspective, age may increase the rate of prion protein conversion: the etiological agent of prion diseases, including CJD^6,7,29^. Also, in our study population, women experienced higher CJD-associated deaths and incidence than men, although it has been generally considered that there is no gender predilection for CJD^12^. A possible explanation for the differences in CJD according to sex may be postmenopausal decreased serum oestrogen levels, which could facilitate the maintenance of cellular pathological prion protein^30^. Hence, the cellular mechanism underlying prion diseases also suggests that the incidence and mortality of sCJD would increase in ageing populations.

Considering that dementia has been reported in almost all cases of CJD in Japan^28^, the increasing incidence of CJD is even more alarming. According to the latest WHO statistics, about 50 million people in the world are estimated to have dementia^31^, imposing a substantial worldwide socioeconomic burden; the global social costs due to dementia were estimated to be 1.1% of the global gross domestic product in 2015^32^. Approximately 4.7 million people were living with dementia in Japan in 2015. This figure is projected to rapidly increase to 7 million people by 2025^33^. Recognising the pressing global concern about dementia, the G20 Health Ministers stressed the importance of developing national strategies to improve the quality of care and life of the patients with dementia as well those of their caregivers^17^. It should further be noted that Japanese patients with CJD were reported to have a longer life expectancy (20.9 months on average) following diagnosis than those from other countries (approximately 6 months as noted above)^28^, which would pose additional socioeconomic impacts on the patients’ caregivers if the mortality and incidence rates continue to rise at the current rates^34^. While CJD still remains a rare disease, the trends identified by the present study warrant attention by public health authorities and indicate the need for effective policy measures to mitigate the disease burden of CJD, from caregiver’s perspectives in particular.

While this study benefits from being the first to use national vital statistics and surveillance data to perform a joinpoint regression analysis of trends in CJD-associated incidence and mortality in Japan, it is subject to several limitations. First, as we analysed the causes of death based on the information provided by death certificates, we may have underestimated the CJD-associated death rates. Second, the rate of CJD incidences could have been further underestimated due to the voluntary nature of the CJD surveillance. Third, we used a specific ICD-10 code (A81.0 “Creutzfeldt–Jakob disease”) from the vital statistics to obtain our data, which made it impossible to determine the accuracy of the recorded causes of death. Specifically, the patients with CJD may have a number of complications, such as infections, heart failure, and respiratory failure, common in the older populations. Hence, the attribution of the cause of death to CJD may have been recorded inappropriately in some cases. Despite the limitations, our present study underscores the significantly increasing trends in CJD-associated mortality and incidence across a 10-year period in the era of ageing.

In conclusion, we revealed the increasing trends of CJD-associated incidence and mortality using joinpoint regression analysis. The extent of increase was greater among individuals older than 70 years of age; hence, the increased rated of CJD-associated incidence and mortality may not only be attributable to increased disease awareness but also be attributable to the increasingly ageing population. The severe socioeconomic burdens on caregivers imposed by CJD-induced dementia also warrant the attention of policymakers and stress the need for a mitigative action plan with particular focus on preparations to handle an increase in the prevalence of dementia.

## Methods

### Data source

The data were acquired from two different sources. First, we examined the Japanese Vital Statistics data from 2005 to 2014 to obtain data on CJD-associated mortality^35^. In Japan, death certificates are issued by physicians and gathered by the Ministry of Health, Labour and Welfare Japan (MHLW). The data on patients’ basic demographics are anonymized and subsequently coded to convey the underlying causes of death in accordance with the designations used in the *International Classification of Diseases, 10th revision* (ICD-10). CJD-associated mortality was defined using the ICD-10 codesA81 (A81.0), named “Creutzfeldt–Jakob disease,” according to the method used by previous studies^12,20^. Thus, in the present study, data on CJD-associated mortality were of the same type as those used in previous studies. Population data were obtained from the 2005 and 2010 censuses. Since the population census is only performed every 5 years in Japan, the government provides projected data for the intervals between census years (2006–2009, 2011–2014). All the data used in the present study, therefore, corresponded to official Japanese statistics, as managed by the Statistics Bureau, Ministry of Internal Affairs and Communications^36^.

Second, we obtained the CJD incidence rates from 2005 to 2014 from the registry data of CJD administered by the Surveillance Committee, which is funded by the MHLW^28^. Although the data is considered reliable because the Surveillance Committee convenes biannually to thoroughly confirm whether the reported cases meet the WHO’s diagnostic criteria for CJD, due to the voluntary nature of the case reports to the Committee, a few years may elapse before the incidence data correspond to the actual numbers.

Data of patients aged over 50 years were stratified by age and sex to calculate crude and age-adjusted mortality rates per 1,000,000 individuals. Due to the lack of sex-stratified incidence data, the age-adjusted incidence rates were calculated en bloc. The age groups were categorised as follows: 50–59 years, 60–69 years, 70–79 years, and ≥ 80 years. All patients aged less than 50 years were excluded from the analysis due to the negligible number of CJD-associated deaths in the age group.

### Statistical analyses and data processing

A joinpoint regression model was implemented with the Joinpoint Regression Program (version 4.8.0.1, April 2020, Statistical Research and Applications Branch, National Cancer Institute, USA). The APCs between trend-change points were determined with 95% confidence intervals (CI). To calculate the age-adjusted rates of CJD-associated mortality and incidence, we used the direct age-standardisation method with the 2005 Japanese population split into 10-year age groups as the standard population. To compare the differences in mortality and incidence trends between population subgroups, the average annual percentage change AAPC was computed for the entire period. The threshold for statistical significance was defined as *p* value < 0.05, which indicated the level at which the slope differed from zero.

### Ethical approval

We used the publicly available data published by the Ministry of Health, Labour and Welfare and the Statistics Bureau of the Ministry of Internal Affairs and Communications Japan. The study was approved by the institutional review board of Okayama University Hospital with a waiver for informed consent because the study intended to retrospectively analyse open data (No. 1910-009). All research methods were performed in accordance with relevant guidelines and regulations.

## Supplementary information

Supplementary Information.

## Data Availability

The datasets generated and analysed during the current study are available from the corresponding author on reasonable request.
