# Supplementary material for: A nationwide trend analysis in the incidence and mortality of Creutzfeldt–Jakob disease in Japan between 2005 and 2014
Source: Sci Rep. 2020 Sep 23;10:15509. doi: 10.1038/s41598-020-72519-0 (PMC7511945; doi:10.1038/s41598-020-72519-0)
Supplement: Supplementary file 1 — Supplementary Information. [file 41598_2020_72519_MOESM1_ESM.docx]

**A nationwide trend analysis in the incidence and mortality of Creutzfeldt-Jakob disease in Japan between 2005 and 2014**

Yoshito Nishimura^1*^, Ko Harada^1^, Toshihiro Koyama^2^, Hideharu Hagiya^1^, Fumio Otsuka^1, +^

^1^Department of General Medicine, Okayama University Graduate School of Medicine, Dentistry and Pharmaceutical Sciences, Okayama, 7008558, Japan

^2^Department of Pharmaceutical Biomedicine, Okayama University Graduate School of Medicine, Dentistry, and Pharmaceutical Sciences, Okayama, 7008558, Japan

***Corresponding author**

Yoshito Nishimura, M.D., Ph.D.

Department of General Medicine, Okayama University Graduate School of Medicine, Dentistry and Pharmaceutical Sciences

2-5-1 Shikata-cho, Kita-ku, Okayama 700-8558, Japan

Phone: +81-86-235-7342 Fax: +81-86-235-7345　E-mail: [nishimura-yoshito@okayama-u.ac.jp](mailto:nishimura-yoshito@okayama-u.ac.jp)
**Supplementary Table 1. Crude mortality rates of Creutzfeldt-Jakob Disease per 1,000,000 persons, 2005-2014**

| **Age(years)** | **2005** | **2006** | **2007** | **2008** | **2009** | **2010** | **2011** | **2012** | **2013** | **2014** |
| --- | --- | --- | --- | --- | --- | --- | --- | --- | --- | --- |
| ≥50 |  |  |  |  |  |  |  |  |  |  |
| Overall | 2.8 | 3.1 | 3.0 | 3.6 | 2.9 | 3.7 | 3.7 | 4.1 | 4.3 | 4.1 |
| Men | 2.9 | 2.6 | 2.8 | 3.7 | 3.0 | 3.5 | 3.9 | 3.5 | 4.2 | 4.3 |
| Women | 2.8 | 3.5 | 3.2 | 3.5 | 2.9 | 3.8 | 3.6 | 4.6 | 4.3 | 4.0 |
| 50-59 |  |  |  |  |  |  |  |  |  |  |
| Overall | 0.8 | 0.9 | 0.5 | 1.1 | 0.8 | 1.3 | 1.1 | 1.4 | 1.2 | 0.8 |
| Men | 0.9 | 1.0 | 0.5 | 1.4 | 0.7 | 1.1 | 1.5 | 0.9 | 1.2 | 1.0 |
| Women | 0.6 | 0.8 | 0.5 | 0.8 | 0.8 | 1.6 | 0.6 | 1.9 | 1.3 | 0.6 |
| 60-69 |  |  |  |  |  |  |  |  |  |  |
| Overall | 3.1 | 3.5 | 3.2 | 3.3 | 2.6 | 3.0 | 3.2 | 2.8 | 3.5 | 3.6 |
| Men | 3.2 | 2.6 | 3.2 | 3.2 | 2.4 | 3.8 | 3.0 | 2.3 | 3.5 | 4.0 |
| Women | 2.9 | 4.3 | 3.2 | 3.4 | 2.8 | 2.2 | 3.4 | 3.3 | 3.5 | 3.3 |
| 70-79 |  |  |  |  |  |  |  |  |  |  |
| Overall | 6.0 | 5.5 | 5.9 | 6.8 | 5.1 | 6.6 | 6.2 | 7.4 | 6.9 | 6.6 |
| Men | 6.0 | 5.1 | 5.4 | 6.3 | 5.9 | 5.0 | 7.1 | 7.1 | 7.4 | 7.0 |
| Women | 6.0 | 5.8 | 6.4 | 7.2 | 4.4 | 8.0 | 5.5 | 7.6 | 6.5 | 6.3 |
| 80- |  |  |  |  |  |  |  |  |  |  |
| Overall | 2.4 | 4.0 | 4.1 | 4.8 | 4.8 | 5.4 | 6.1 | 6.4 | 7.0 | 6.5 |
| Men | 2.4 | 3.2 | 4.2 | 7.2 | 5.6 | 6.9 | 6.9 | 6.5 | 7.2 | 7.4 |
| Women | 2.3 | 4.4 | 4.0 | 3.6 | 4.4 | 4.6 | 5.7 | 6.3 | 6.9 | 6.0 |
